# Transcription Factors Leading to High Expression of Neuropeptide L1CAM in Brain Metastases from Lung Adenocarcinoma and Clinical Prognostic Analysis

**DOI:** 10.1155/2021/8585633

**Published:** 2021-12-30

**Authors:** Xu Feng, Ning Guan, Enshi Xu, Ye Miao, Chenguang Li

**Affiliations:** ^1^Department of Neurosurgery, The First Affiliated Hospital of Jinzhou Medical University, China; ^2^Department of Neurointervention, The First Affiliated Hospital of Jinzhou Medical University, China; ^3^Department of General Surgery, The First Affiliated Hospital of Jinzhou Medical University, China

## Abstract

**Background:**

There is a lack of understanding of the development of metastasis in lung adenocarcinoma (LUAD). This study is aimed at exploring the upstream regulatory transcription factors of L1 cell adhesion molecule (L1CAM) and to construct a prognostic model to predict the risk of brain metastasis in LUAD.

**Methods:**

Differences in gene expression between LUAD and brain metastatic LUAD were analyzed using the Wilcoxon rank-sum test. The GRNdb (http://www.grndb.com) was used to reveal the upstream regulatory transcription factors of L1CAM in LUAD. Single-cell expression profile data (GSE131907) were obtained from the transcriptome data of 10 metastatic brain tissue samples. LUAD prognostic nomogram prediction models were constructed based on the identified significant transcription factors and L1CAM.

**Results:**

Survival analysis suggested that high L1CAM expression was negatively significantly associated with overall survival, disease-specific survival, and prognosis in the progression-free interval (*p* < 0.05). The box plot indicates that high expression of L1CAM was associated with distant metastases in LUAD, while ROC curves suggested that high expression of L1CAM was associated with poor prognosis. FOSL2, HOXA9, IRF4, IKZF1, STAT1, FLI1, ETS1, E2F7, and ADARB1 are potential upstream transcriptional regulators of L1CAM. Single-cell data analysis revealed that the expression of L1CAM was found significantly and positively correlated with the expression of ETS1, FOSL2, and STAT1 in brain metastases. L1CAM, ETS1, FOSL2, and STAT1 were used to construct the LUAD prognostic nomogram prediction model, and the ROC curves suggest that the constructed nomogram possesses good predictive power.

**Conclusion:**

By bioinformatics methods, ETS1, FOSL2, and STAT1 were identified as potential transcriptional regulators of L1CAM in this study. This will help to facilitate the early identification of patients at high risk of metastasis.

## 1. Introduction

Lung cancer is a malignant tumor with increasing incidence and high mortality rates worldwide in recent years [1]. Non-small-cell lung cancer (NSCLC) accounts for approximately 85% of lung cancers, and lung adenocarcinoma (LUAD) accounts for 60% of diagnosed NSCLC, making it the most common type of NSCLC [2]. LUAD is a malignant tumor of the glandular epithelium. Current studies suggest that most LUADs progress through a sequence of atypical adenomatous hyperplasia (AAH), adenocarcinoma in situ (AIS), microinvasive adenocarcinoma (MIA), and finally invasive adenocarcinoma (IA). However, the exact mechanism of disease progression remains unclear [3].

Tumor infiltration and metastasis are important factors in the low overall survival of patients with LUAD. [4] Most patients with LUAD are diagnosed at an advanced stage or have distant metastases. The brain is one of the most common sites of hematogenous metastases of LUAD, and metastasis at this site is associated with high morbidity and mortality [5]. Approximately 10% to 20% of non-small-cell lung cancer patients have brain metastases at the initial diagnosis, and the majority are LUAD patients [5, 6]. Additionally, about 40% to 60% of LUAD patients develop brain metastases during treatment [7]. According to the latest prognostic assessment model for brain metastases from lung cancer, the median survival of LUAD patients with brain metastases is approximately 15 months, which significantly affects patient prognosis [8]. Although TNM staging plays an important role in assessing the prognosis of patients with LUAD, some patients with similar staging and identical treatment courses had a significantly different prognosis.

L1 cell adhesion molecule protein (L1CAM) is a transmembrane glycoprotein with a molecular weight of 200 to 220 kDa and is a member of the immunoglobulin superfamily of cell adhesion molecules, which plays an important role in the development and regeneration of neural tissue [9, 10]. As an adhesion molecule, L1CAM can increase the migration and invasion abilities of tumor cells, specifically by promoting tumor cells to cross the endothelium, invade the basement membrane, and metastasize to other sites, thus playing an important role in tumor development and bloodstream metastasis [11]. In recent years, L1CAM has been found to be highly expressed in many tumor cell lines and tumor tissues—for example, in glioblastoma, metastatic brain tumors, endometrioid adenocarcinoma, colorectal cancer, and lung cancer [12–16]. Its high expression often indicates a poor prognosis, and it is thus a valuable diagnostic or prognostic marker; in addition, it may be a new target for cancer therapy [12, 13, 17–23].

In recent years, with the boom in single-cell technology, a large number of single-cell datasets have been assayed for the analysis of gene regulatory networks in individual cells [14–16]. A large number of single-cell data mining studies have been carried out, contributing to the flourishing of research in the field of tumor microenvironment and cell development [24–27]. A study published in Nat Commun in 2020 reveals the transcriptome signature of LUAD brain metastases [28]. This dataset was used in this study to explore the potential regulatory network of L1CAM.

There remains a lack of understanding of the development of LUAD metastases resulting from L1CAM. We explored the potential mechanisms of L1CAM-related LUAD metastasis, and our results may provide new targets and ideas for the treatment of LUAD patients.

## 2. Materials and Methods

### 2.1. Data Retrieval

RNAseq data and clinical information were downloaded from the Cancer Genome Atlas (TCGA) (https://portal.gdc.cancer.gov/) LUAD project. The R package DESeq2 (version 1.26.0) was used for the variance analysis. Single-cell expression profile data were obtained from the transcriptome data of 10 metastatic brain tissue samples; information on single-cell annotations was downloaded from the same Gene Expression Omnibus (GEO) dataset GSE131907 [28]. Differences in gene expression between these two datasets were analyzed using the Wilcoxon rank-sum test, respectively.

### 2.2. Enrichment Analysis

Enrichment analysis is an important means of demonstrating gene function. Gene oncology (GO) and Kyoto Encyclopedia of Genes and Genomes (KEGG) enrichment analyses were done with the clusterProfiler package (version 3.14.3) of R software (version 3.6.3). The http://org.Hs.eg.db (version 3.10.0) package was used for ID conversion. *p* < 0.05 was defined as statistically different. In addition, we performed gene set enrichment analysis (GSEA), an analysis method for genome-wide expression profiling microarray data that compares genes to a predefined gene set and allows for an understanding of the expression status of target genes in a specific set of functional genes, was performed. False discovery rate < 0.25 and adjusted *p* value < 0.05 were defined as significant enrichment.

### 2.3. Immune Infiltration and StromalScore Analysis

Different immune cells play different roles in tumorigenesis, and the composition of immune cells varies from tumor to tumor [29]. Therefore, quantitative immune infiltration analysis of different types of immune cells is often carried out in the study of tumor mechanisms. The specific methods of immune infiltration analysis were similar to those of previous studies. Cell markers were derived from previous studies [30]. The analysis for immunoinfiltration was performed on the retrieved dataset using ssGSEA, a built-in algorithm of the GSVA package in R. Immune infiltration and StromalScore analysis were performed using the CIBERSORT and ESTIMATE algorithms to calculate the degree of immune cell infiltration and immune, mesenchymal, and tumor purity in TCGA. The differences in immune cell infiltration and tumor purity between subtypes were further compared [31].

### 2.4. Prediction of Transcription Factors

Transcription factors and their downstream target genes form a gene regulatory network that plays a key role in regulating gene expression. The Gene Regulatory Network database (GRNdb) (http://www.grndb.com/) is a freely accessible database that provides a reliable way to explore gene expression profiles, correlations, and expression levels [32]. In this study, the GRNdb was used to reveal the upstream regulatory transcription factors of L1CAM in LUAD.

### 2.5. Construction of a LUAD Prognostic Nomogram Prediction Model

The nomogram is a visualization of the regression model results, which can be easily and quickly applied to the clinical assessment of patient prognosis [33–36]. The Cox regression analysis included risk genes for LUAD to construct a lung cancer risk prediction model, which was then used to predict the probability of survival at 1, 2, and 3 years for LUAD patients. The time-dependent receiver operating characteristic curve (ROC) and the area under the curve (AUC) values at 3 and 5 years were used to evaluate the independent predictive ability of the nomogram factors. In addition, calibration curves were plotted to check the accuracy of the nomogram model.

### 2.6. Statistical Analysis

All RNAseq data in fragments per kilobase of transcript per million mapped reads (FPKM) format were converted to TPM (transcripts per million reads) format and log2 transformed. Statistical analysis of survival data was done with the survivor R package, and visualization was done using the survminer R package. Correlation analysis was performed using the Spearman method. The ggplot2 package (version 3.3.3) of the R software was used for data visualization [37].

## 3. Results

### 3.1. Functional Features of L1CAM in LUAD

**Figure 1:**
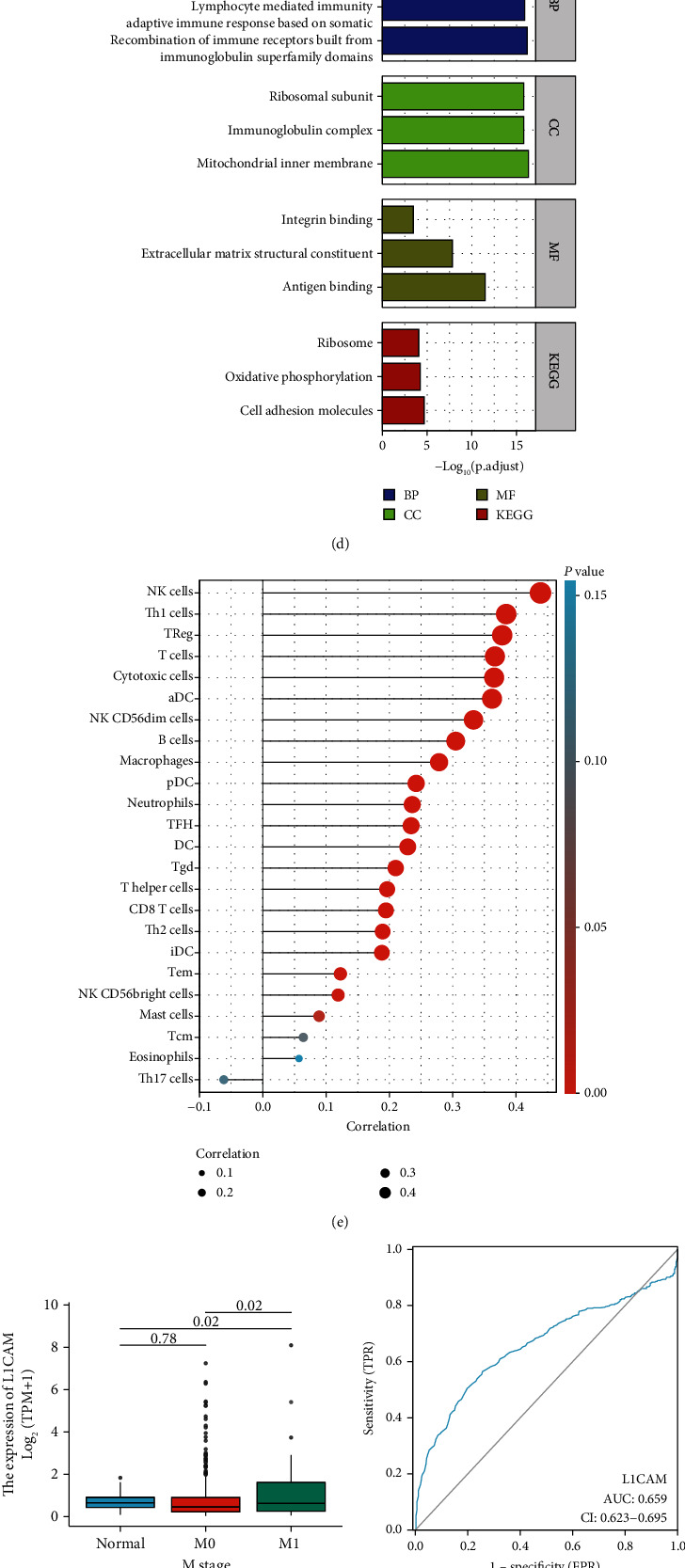
Functional features of L1CAM in LUAD. Survival curves of high and low L1CAM expressions in relation to LUAD overall survival (OS) (a), disease-specific survival (DSS) (b), and progression-free interval (PFI) (c); enrichment analysis associated with differential expression of L1CAM (d). Lollipop plot of L1CAM expression in relation to the degree of immune cell infiltration (e). Boxplot of differential expression of L1CAM in different M stages (f). Receiver operating characteristic curves for assessing the predictive power of L1CAM expression on survival (AUC = 0.659, 95% CI 0.623-0.695) (g). Boxplot showing the relationships between high and low L1CAM expressions, T cell enrichment score, and StromalScore (h, i). GSEA analysis of the enriched pathway associated with L1CAM expression (j).

Survival analysis suggested that high L1CAM expression is significantly associated with overall survival, disease-specific survival, and poor prognosis at the progression-free interval (*p* < 0.05; Figures 1(a)–1(c)). In the TCGA-LUAD dataset, the enriched genes associated with high L1CAM expression per group were mainly in recombination of immune receptors built from immunoglobulin superfamily domains for biological process (BP), mitochondrial inner membrane for cellular component (CC), and antigen-binding for molecular function (MF) as shown in Figure 1(d). Meanwhile, KEGG pathway analysis showed that the genes associated with high L1CAM expression were mainly enriched in the cell adhesion molecule pathway (Figure 1(d)). The high expression of L1CAM was significantly correlated with the enrichment of NK cells, Th1 cells, Treg, T cells, cytotoxic cells, ADC, NK CD56dim cells, and B cells by immunoinfiltration analysis (Figure 1(e)). High expression of L1CAM was also significantly and positively correlated with the enrichment score of T cells and StromalScore (Figures 1(h)–1(i)). The boxplot of differential expression of L1CAM in M0 versus M1 was shown in Figure 1(f), suggesting that high L1CAM expression is associated with distant metastasis, while the derived ROC curves suggest that high expression of L1CAM is associated with poor prognosis in LUAD. GSEA enrichment analysis suggested that the functions of L1CAM were significantly enriched in KEGG terms related to ECM receptor interaction, hematopoietic cell lineage, natural killer cell-mediated cytotoxicity, and pathways in cancer (Figure 1(j)).

### 3.2. Potential Upstream Regulatory Targets of L1CAM in LUAD Bloodstream Metastases

**Figure 2:**
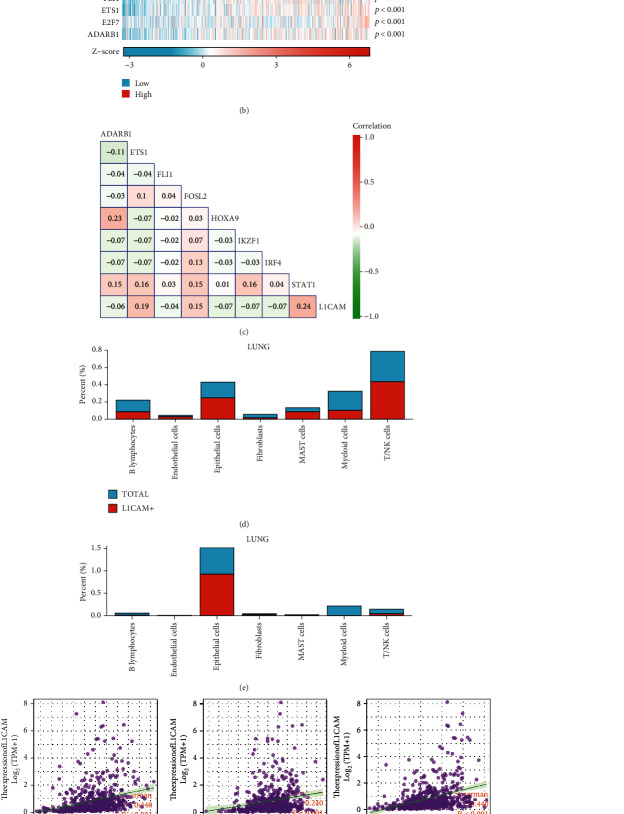
Potential upstream regulatory targets of L1CAM and single-cell analysis. Predicted potential upstream transcriptional regulators of L1CAM in LUAD based on Gene Regulatory Network database (GRNdb) (a). Heat map analysis of the correlation of the predicted transcription factors with L1CAM expression in the TCGA-LUAD dataset (b). Correlation of these transcription factors with L1CAM expression in the GSE131907 LUAD brain metastasis malignancy cell dataset (c). Analysis of the proportion of L1CAM-positive cells in LUAD (d) and LUAD cerebrovascular metastases (e). Correlation analysis of L1CAM expression and selected transcription factors ETS1, FOSL2, and STAT1 (f). Survival curves for the relationship between high and low transcription factor expressions and LUAD overall survival (g–i): ETS1 (g), FOSL2 (h), and STAT1 (i).

In this study, the GRNdb was used to reveal the upstream regulatory transcription factors of L1CAM. The predicted potential upstream transcriptional regulators are FOSL2, HOXA9, IRF4, IKZF1, STAT1, FLI1, ETS1, E2F7, and ADARB1. Heat map analysis of the correlation between these transcription factors and L1CAM expression in the TCGA-LUAD dataset suggested that all these transcriptional regulators were significantly and positively correlated with L1CAM expression (*p* < 0.001). The correlation analysis of these transcription factors with L1CAM expression in the GSE131907 LUAD brain metastasis malignancy cell dataset, meanwhile, is shown in Figure 2(c). Of the above regulators, ETS1, FOSL2, and STAT1 were found to be key transcriptional regulators in LUAD brain metastases.

Analysis of the proportion of L1CAM-positive cells in LUAD and LUAD cerebrovascular metastases suggested that L1CAM was significantly more highly expressed in the brain tissue at the metastasis site (Figure 2(e)). Scatter plot correlation analysis suggested that the expression of L1CAM was significantly and positively correlated with the expression of ETS1, FOSL2, and STAT1 (Figure 2(f)). Survival analysis suggested that high expression of FOSL2 and STAT1 was significantly associated with poor prognosis of LUAD (*p* < 0.05), and high expression of ETS1 was also potentially correlated with poor prognosis of LUAD (*p* = 0.053) (Figure 2(g)).

### 3.3. KEGG Analysis of the Upstream Regulatory Targets of L1CAM

**Figure 3:**
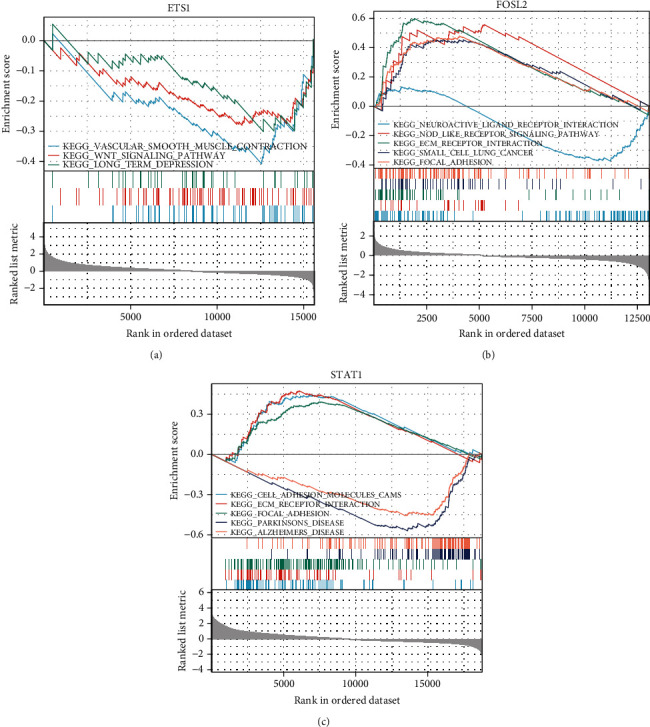
Enrichment analysis of the KEGG pathway for upstream regulatory targets of L1CAM. The analyses are shown for ETS1 (a), FOL2 (b), and STAT1 (c).

To further clarify the potential functions of ETS1, FOSL2, and STAT1 in LUAD transfer, we performed KEGG analysis on these regulators using GSEA (Figure 3). The functions of ETS1 are mainly related to vascular smooth muscle contraction, the Wnt signaling pathway, and long-term depression (Figure 3(a)). The functions of FOSL2 are mainly related to neuroactive ligand-receptor interaction, nod-like receptor signaling pathway, ECM receptor interaction, small cell lung cancer, and focal adhesion (Figure 3(b)). The functions of STAT1 are mainly related to cell adhesion molecules (CAMs), ECM receptor interaction, focal adhesion, Parkinson's disease, and Alzheimer's disease (Figure 3(c)).

### 3.4. LUAD Prediction Model Constructed Based on L1CAM, ETS1, FOSL2, and STAT1

**Figure 4:**
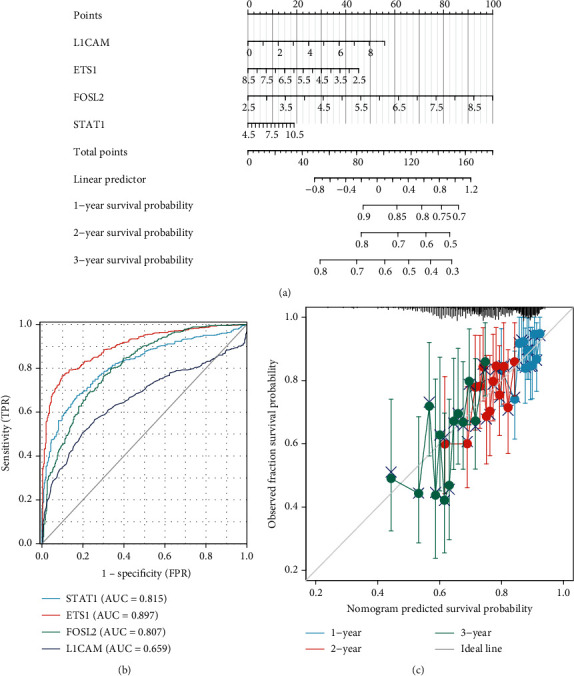
Construction of a prognostic nomogram prediction model for LUAD. Prognostic nomogram prediction model for LUAD based on L1CAM, ETS1, FOSL2, and STAT1 (a). ROC curves of L1CAM, ETS1, FOSL2, and STAT1 associated with LUAD (b). Calibration curves for this nomogram to predict the probability of survival at 1, 2, and 3 years for LUAD patients (c). “Observed fraction survival probability” means the actual observed survival rate. “Nomogram predicted survival probability” refers to the survival rate that was predicted.

Based on the strong correlations found in our analysis, L1CAM, ETS1, FOSL2, and STAT1 were thus used to construct the LUAD prognostic nomogram prediction model. Nomogram models predicting the probability of survival at 1, 2, and 3 years postdiagnosis for LUAD patients were constructed (Figure 4(a)). The total score was obtained by summing the scores for each item of information about the LUAD patient, and the probability of survival was given as a total score on the scale. The ROC curves of L1CAM, ETS1, FOSL2, and STAT1 associated with LUAD are shown in Figure 4(b). The results suggest that L1CAM, ETS1, FOSL2, and STAT1 have predictive power for LUAD prognosis. The calibration curve of the nomogram model was also shown in Figure 4(c), suggesting that the nomogram has good predictive power.

## 4. Discussion

As a common pathological type of lung cancer, the incidence and mortality rates of LUAD are on the rise [3, 38, 39]. Despite new advances in research into the diagnosis and clinical management of LUAD, the average 5-year survival rate for patients with LUAD is only 15%, and the related deaths account for nearly 30% of cancer-related deaths worldwide [40, 41]. The mechanism of LUAD brain metastasis is still unclear, which hinders early detection and interruption of metastasis. Therefore, the active search for biological markers of LUAD brain metastases is of great clinical importance for the early warning.

The TCGA database was used to analyze differential genes in LUAD patients with GO and KEGG enrichment analyses in this study. The GRNdb database was used to reveal the upstream regulatory transcription factors of L1CAM in LUAD. Based on this, L1CAM, ETS1, FOSL2, and STAT1 were incorporated to construct a prognostic nomogram prediction model to assess the risk of LUAD metastasis.

The functions of specific sets of genes in the tumor system are increasingly being revealed [42, 43]. Previous studies have revealed that L1CAM is involved in the malignant phenotype of tumors through various signaling pathways, the more classic being the Wnt/*β*-catenin/TCF pathway that promotes tumor metastasis, but also by activating the Ras/Raf/Mek/Erk signaling pathway to promote epithelial-mesenchymal transition [44, 45]. Inhibition of L1CAM expression has been shown to reduce the motility and invasiveness of NSCLC cells *in vitro* and tumorigenesis and distant metastasis *in vivo*.^18^ Similar to previous studies, the present study found that high expression of L1CAM was associated with distant LUAD metastases. In addition, L1CAM was significantly more highly expressed in metastases from brain tissue than lung tissue in LUAD, suggesting that it could be a potential marker of LUAD metastasis.

Enrichment analysis showed that the genes associated with high L1CAM expression were mainly enriched in recombination of immune receptors built from immunoglobulin superfamily domains in BPs, mitochondrial inner membrane in CCs, and antigen-binding in MFs. In addition, high L1CAM expression was significantly associated with the enrichment of NK cells, Th1 cells, Tregs, T cells, cytotoxic cells, ADC, NK CD56dim cells, and B cells, suggesting that L1CAM may participate in the progression of LUAD by regulating the role of immune cells in the microenvironment. Among the pathways, cell adhesion molecules were enriched. This suggests that the pathway may be involved in the development of LUAD and affect patient prognosis.

Many different transcriptional regulators regulate L1CAM, and among these, ETS1, FOSL2, and STAT1 were found to be significantly correlated with expression in LUAD. E26 transformation-specific-1, or ETS1, is a member of the ETS transcription factor family and is involved in the degradation of extracellular matrix proteins and cellular hypoxia tolerance through self-regulation and bypass regulation [46]. It can activate or repress the transcription of certain target genes and is involved in the growth and differentiation of a wide range of immune cells and the regulation of the expression of many cytokines [47, 48]. The ETS1 signaling pathway was found to promote tumor cell migration, invasion, and secretion of matrix metalloproteinases (MMPs), which was closely associated with lymph node metastasis and distant metastasis in patients with lung, colon, ovarian, and breast cancers [49–52]. As in previous studies, we found that ETS1 was one of the key transcriptional regulators of brain metastasis in LUAD, and the expression of L1CAM was significantly and positively correlated with the expression of ETS1. KEGG analysis showed that the function of ETS1 was enriched in pathways related to immune cell maintenance and renewal, suggesting that ETS1 may influence the development of LUAD through relevant pathways. High expression of ETS1 was also potentially correlated with poor prognosis in LUAD, and the ROC curve suggested that ETS1 had some predictive power for lung cancer prognosis. However, to our knowledge, no other studies have identified a potential link between ETS1 and L1CAM. In the present study, ETS1 was found to function as a potential transcription factor for L1CAM in LUAD brain metastases.

FOS-related antigen 2 (FOSL2) belongs to the AP-1 family of transcription factors and plays an important role in tumor proliferation and cell cycle regulation [53]. FOSL2 was found to be aberrantly expressed in non-small-cell lung cancer, ovarian cancer, liver cancer, and other malignant tumors and is involved in the growth and metastasis of tumor cells as a prooncogene [54–56]. In this study, FOSL2 was found to be an upstream regulatory transcription factor of L1CAM in LUAD, and the expression of L1CAM was significantly and positively correlated with that of FOSL2. KEGG analysis showed that the function of FOSL2 was mainly enriched in the neuroactive ligand-receptor signaling pathway, Nod-like receptor signaling, ECM receptor interaction, small cell lung cancer, and focal adhesion, suggesting that FOSL2 may play a role in LUAD through these pathways. The ROC curves suggest that FOSL2 has a predictive power for LUAD prognosis, similar to the findings of Wang et al., who found that FOSL2 promoted TGF-*β*1-induced migration in NSCLC, and that patients with higher FOSL2 expression had a significantly higher risk of premature death [54].

The signal transducer and activator of transcription (STAT) family is a group of proteins that have transcriptional activity and transmit signals from the cell membrane into the nucleus, thereby activating gene transcription. The most important functions lie in activating the body's immune response and the regulation of cell proliferation and transformation [57, 58]. STAT1 was the first member of the STAT family to be identified and is commonly regarded as a tumor suppressor protein in malignant tumors such as breast cancer, melanoma, and leukemia [59–61]. However, the role of STAT1 in the progression of different tumors remains controversial. It has been suggested that the IFN/STAT1 signaling pathway may promote the growth of tumor cells [62, 63]. STAT1 is overexpressed in specific cellular environments and is associated with poor prognosis in cancer patients [64]. In this study, STAT1 was found to be an upstream regulatory transcription factor of L1CAM in LUAD, and the expression of L1CAM was significantly and positively correlated with that of STAT1. KEGG analysis showed that high STAT1 expression was associated with the enrichment of cell adhesion and neural function pathways. High expression of STAT1 was significantly associated with poor prognosis in LUAD. The present study revealed that STAT1 may act as a potential transcriptional regulator of L1CAM in the brain metastasis of LUAD. The relevance of STAT1 to L1CAM has been revealed in several previous studies. For example, STAT1 and L1CAM expressions were found to be jointly downregulated in diabetes-related skin disorders [65]. In colorectal cancer, L1CAM caused upregulation of clusterin expression in cancer cells as a result of the transactivating effect of STAT1 on clusterin [66]. Previous studies have also suggested that L1CAM may activate STAT1, but only revealed a correlation between them. The interaction between the two has yet to be verified by further experiments [67].

Thus, high L1CAM levels may indicate immune response abnormalities, tumorigenesis, and the dysregulation of neuron functions. High L1CAM expression was significantly associated with poor prognosis for OS, DSS, and PFI in LUAD patients. The ROC curves suggest that L1CAM has some predictive power for LUAD prognosis. This is similar to the findings of Yu et al., who concluded that L1CAM was a predictor of PFS in non-small-cell lung cancer patients and that positive expression of L1CAM suggested a poorer survival outcome [68]. A nomogram model was thus constructed using L1CAM, ETS1, FOSL2, and STAT1 to predict the survival probability of LUAD patients at 1, 2, and 3 years. The calibration curves suggest that the nomogram has a good predictive ability and is expected to be a valid indicator for assessing the prognosis of LUAD patients.

Based on multiple validation of TCGA, GRNdb, and GSE131907 scRNA datasets, ETS1, FOSL2, and STAT1 were identified as potential transcriptional regulators of L1CAM. However, as this study was based on the TCGA database and the mRNA expression data of GSE131907, its race specificity is obvious and its applicability to other species remains to be further investigated. In addition, the genes used in this study were taken from public databases, and the genes used in the model construction were only statistically significant associations, and their correlation with etiology and clinical immunotherapy outcomes cannot be confirmed at this time. Further experimental validation of the clinical value of the model in this study is needed.

## 5. Conclusion

By bioinformatics methods, ETS1, FOSL2, and STAT1 were identified as potential transcriptional regulators of L1CAM in this study. The nomogram prediction model based on L1CAM, ETS1, FOSL2, and STAT1 can be used as an intuitive and noninvasive quantitative tool to predict the risk of LUAD metastasis. This would facilitate the early identification of patients at high risk of metastasis for early clinical intervention and guide individualized treatment planning to improve the prognosis of LUAD patients.

## Data Availability

RNAseq data and clinical information were from the TCGA (https://portal.gdc.cancer.gov/) LUAD project; information on single-cell annotations was downloaded in GSE131907.

## Abbreviations

AAH: Atypical adenomatous hyperplasia
AIS: Adenocarcinoma in situ
ETS1: E26 transformation-specific-1
FOSL2: Fos-related antigen 2
GEO: Gene Expression Omnibus
IA: Invasive adenocarcinoma
L1CAM: L1 cell adhesion molecule
LUAD: Lung adenocarcinoma
MIA: Microinvasive adenocarcinoma
NSCLC: Non-small-cell lung cancer
STAT1: Signal transducer and activator of transcription 1
TCGA: The Cancer Genome Atlas.

## References

[1] Chen W., Zheng R., Baade P. D. (2016). cancer statistics in China, 2015. *CA: a Cancer Journal for Clinicians*.

[2] Wu C., Li X., Zhang D. (2018). IL-1*β*-mediated up-regulation of WT1D via miR-144-3p and their synergistic effect with NF-*κ*B/COX-2/HIF-1*α* pathway on cell proliferation in LUAD. *Cellular Physiology and Biochemistry*.

[3] Travis W. D., Brambilla E., Nicholson A. G. (2015). The 2015 World Health Organization classification of lung tumors: impact of genetic, clinical and radiologic advances since the 2004 classification. *Journal of Thoracic Oncology*.

[4] Peters S., Adjei A. A., Gridelli C., Reck M., Kerr K., Felip E. E. G. W. G. (2012). Metastatic non-small-cell lung cancer (NSCLC): ESMO Clinical Practice Guidelines for diagnosis, treatment and follow-up^†^. *Annals of Oncology*.

[5] Jackman D. M., Zhang Y., Dalby C. (2017). Cost and survival analysis before and after implementation of Dana-Farber clinical pathways for patients with stage IV non–small-cell lung cancer. *Journal of the Pancreas: JOP*.

[6] Lee J. S., Hong J. H., Won H. S. (2019). The impact of systemic treatment on brain metastasis in patients with non- small-cell lung cancer: A retrospective nationwide population-based cohort study. *Scientific Reports*.

[7] Barnholtz-Sloan J. S., Sloan A. E., Davis F. G., Vigneau F. D., Lai P., Sawaya R. E. (2004). Incidence proportions of brain metastases in patients diagnosed (1973 to 2001) in the metropolitan Detroit cancer surveillance system. *Journal of clinical oncology*.

[8] Arrieta O., Ramírez‐Tirado L. A., Caballé‐Perez E. (2020). Response rate of patients with baseline brain metastases from recently diagnosed non-small cell lung cancer receiving radiotherapy according to *EGFR*, *ALK* and *KRAS* mutation status. *Thoracic cancer*.

[9] Samata B., Takaichi R., Ishii Y. (2020). L1CAM is a marker for enriching corticospinal motor neurons in the developing brain. *Frontiers in Cellular Neuroscience*.

[10] Linneberg C., Toft C. L. F., Kjaer-Sorensen K., Laursen L. S. (2019). L1cam-mediated developmental processes of the nervous system are differentially regulated by proteolytic processing. *Scientific Reports*.

[11] Samatov T. R., Wicklein D., Tonevitsky A. G. (2016). L1CAM: cell adhesion and more. *Progress in Histochemistry and Cytochemistry*.

[12] Altevogt P., Doberstein K., Fogel M. (2016). L1CAM in human cancer: L1CAM in human cancer. *International Journal of Cancer*.

[13] Angiolini F., Cavallaro U. (2017). The pleiotropic role of L1CAM in tumor vasculature. *International journal of molecular sciences*.

[14] Tabula Muris Consortium (2018). Single-cell transcriptomics of 20 mouse organs creates a *Tabula Muris*. *Nature*.

[15] Van de Sande B., Flerin C., Davie K. (2020). A scalable SCENIC workflow for single-cell gene regulatory network analysis. *Nature Protocols*.

[16] Lin W.-W., Xu L. T., Chen Y. S., Go K., Sun C., Zhu Y. J. (2021). Single-cell transcriptomics-based study of transcriptional regulatory features in the mouse brain vasculature. *BioMed Research International*.

[17] Schäfer M. K. E., Altevogt P. (2010). L1CAM malfunction in the nervous system and human carcinomas. *Cellular and Molecular Life Sciences*.

[18] Pace K. R., Dutt R., Galileo D. S. (2019). Exosomal L1CAM stimulates glioblastoma cell motility, proliferation, and invasiveness. *International journal of molecular sciences*.

[19] Wachowiak R., Krause M., Mayer S. (2018). Increased L1CAM (CD171) levels are associated with glioblastoma and metastatic brain tumors. *Medicine*.

[20] Abdel Azim S., Sprung S., Mutz-Dehbalaie I., Fessler S., Zeimet A. G., Marth C. (2017). L1CAM and HER2 expression in early endometrioid uterine cancer. *International Journal of Gynecological Pathology*.

[21] Ganesh K., Basnet H., Kaygusuz Y. (2020). L1CAM defines the regenerative origin of metastasis-initiating cells in colorectal cancer. *Nature cancer*.

[22] Tampakis A., Tampaki E. C., Nonni A. (2020). L1CAM expression in colorectal cancer identifies a high-risk group of patients with dismal prognosis already in early-stage disease. *Acta Oncologica*.

[23] Hai J., Zhu C. Q., Bandarchi B. (2012). L1 cell adhesion molecule promotes tumorigenicity and metastatic potential in mon–small cell lung cancer. *Clinical Cancer Research*.

[24] Kang X., Chen Y., Yi B. (2021). An integrative microenvironment approach for laryngeal carcinoma: the role of immune/methylation/autophagy signatures on disease clinical prognosis and single-cell genotypes. *Journal of Cancer*.

[25] Chen Y., Sun Y., Xu Y. (2021). Single-cell integration analysis of heterotopic ossification and fibrocartilage developmental lineage: endoplasmic reticulum stress effector Xbp1 transcriptionally regulates the notch signaling pathway to mediate fibrocartilage differentiation. *Oxidative Medicine and Cellular Longevity*.

[26] He D., Wang D., Lu P. (2021). Single-cell RNA sequencing reveals heterogeneous tumor and immune cell populations in early-stage lung adenocarcinomas harboring EGFR mutations. *Oncogene*.

[27] Wu J., Qin J., Li L. (2021). Roles of the immune/methylation/autophagy landscape on single-cell genotypes and stroke risk in breast cancer microenvironment. *Oxidative Medicine and Cellular Longevity*.

[28] Kim N., Kim H. K., Lee K. (2020). Single-cell RNA sequencing demonstrates the molecular and cellular reprogramming of metastatic lung adenocarcinoma. *Nature Communications*.

[29] Lois H. (2019). New advances in immunotherapy for prostate cancer. *Life Research*.

[30] Bindea G., Mlecnik B., Tosolini M. (2013). Spatiotemporal dynamics of intratumoral immune cells reveal the immune landscape in human cancer. *Immunity*.

[31] Hänzelmann S., Castelo R., Guinney J. (2013). GSVA: gene set variation analysis for microarray and RNA-Seq data. *BMC Bioinformatics*.

[32] Fang L., Li Y., Ma L., Xu Q., Tan F., Chen G. (2021). GRNdb: decoding the gene regulatory networks in diverse human and mouse conditions. *Nucleic Acids Research*.

[33] Yap W.-K., Shih M. C., Kuo C. (2018). Development and validation of a nomogram for assessing survival in patients with metastatic lung cancer referred for radiotherapy for bone metastases. *JAMA Network Open*.

[34] Zhou C., Shan C., Lai M. (2021). Individualized nomogram for predicting survival in patients with brain metastases after stereotactic radiosurgery utilizing driver gene mutations and volumetric surrogates. *Frontiers in Oncology*.

[35] Kang X., Chen B., Chen Y. S. (2020). A prediction modeling based on SNOT-22 score for endoscopic nasal septoplasty: a retrospective study. *PeerJ*.

[36] Chen Y., Cai Y. X., Kang X. R. (2020). Predicting the risk of sarcopenia in elderly patients with patellar fracture: development and assessment of a new predictive nomogram. *PeerJ*.

[37] Maag J. L. V. (2018). gganatogram: an R package for modular visualisation of anatograms and tissues based on ggplot2. *F1000Res*.

[38] Galvan A., Frullanti E., Anderlini M. (2013). Gene expression signature of non-involved lung tissue associated with survival in lung adenocarcinoma patients. *Carcinogenesis*.

[39] Shukla S., Evans J. R., Malik R. (2017). Development of a RNA-Seq based prognostic signature in lung adenocarcinoma. *Journal of the National Cancer Institute*.

[40] Miller K. D., Siegel R. L., Lin C. C. (2016). Cancer treatment and survivorship statistics, 2016. *CA: a Cancer Journal for Clinicians*.

[41] Jemal A., Siegel R., Xu J., Ward E. (2010). Cancer statistics, 2010. *CA: a Cancer Journal for Clinicians*.

[42] Farhoud Z., Bozorgchenani S. (2021). The mechanism of DDR genes in tumor progression. *Life Research*.

[43] Deng C., Guo H., Yan D., Liang T., Ye X., Li Z. (2021). Pancancer analysis of neurovascular-related NRP family genes as potential prognostic biomarkers of bladder urothelial carcinoma. *BioMed Research International*.

[44] Huszar M., Pfeifer M., Schirmer U. (2010). Up-regulation of L1CAM is linked to loss of hormone receptors and E-cadherin in aggressive subtypes of endometrial carcinomas: L1 in endometrial carcinoma. *The Journal of Pathology*.

[45] Polakis P. (2007). The many ways of Wnt in cancer. *Current Opinion in Genetics & Development*.

[46] Dittmer J., Vetter M., Blumenthal S. G., Lindemann R. K., Kölbl H. (2004). Importance of Ets1 proto-oncogene for breast cancer progression. *Zentralblatt für Gynäkologie*.

[47] Dong Z. (2013). Acetylation of Ets-1 is the key to chromatin remodeling for miR-192 expression. *Science Signaling*.

[48] Dai H., He F., Tsokos G. C., Kyttaris V. C. (2017). IL-23 limits the production of IL-2 and promotes autoimmunity in lupus. *The Journal of Immunology*.

[49] Kosaka T., Miyajima A., Shirotake S. (2010). Ets-1 and hypoxia inducible factor-1*α* inhibition by angiotensin II type-1 receptor blockade in hormone-refractory prostate cancer. *The Prostate*.

[50] Yamaguchi E., Nakayama T., Nanashima A. (2007). Ets-1 proto-oncogene as a potential predictor for poor prognosis of lung adenocarcinoma. *The Tohoku Journal of Experimental Medicine*.

[51] Dejana E., Taddei A., Randi A. (2007). Foxs and Ets in the transcriptional regulation of endothelial cell differentiation and angiogenesis. *Biochimica et Biophysica Acta (BBA) - Reviews on Cancer*.

[52] Li W., Liu C., Zhao C., Zhai L., Lv S. (2016). Downregulation of *β*3 integrin by miR-30a-5p modulates cell adhesion and invasion by interrupting Erk/Ets-1 network in triple-negative breast cancer. *International Journal of Oncology*.

[53] Chen X., Wang Z., Tong F., Dong X., Wu G., Zhang R. (2020). lncRNA UCA1 promotes gefitinib resistance as a ceRNA to target FOSL2 by sponging miR-143 in non-small cell lung cancer. *Molecular Therapy - Nucleic Acids*.

[54] Wang J., Sun D., Wang Y. (2014). FOSL2 positively regulates TGF-*β*1 signalling in non-small cell lung cancer. *PLoS One*.

[55] Li Z., Niu H., Qin Q. (2019). lncRNA UCA1 mediates resistance to cisplatin by regulating the miR-143/FOSL2-signaling pathway in ovarian cancer. *Molecular Therapy - Nucleic Acids*.

[56] Li Z., Liu Y., Yan J. (2020). Circular RNA hsa_circ_0056836 functions an oncogenic gene in hepatocellular carcinoma through modulating miR-766-3p/FOSL2 axis. *Aging*.

[57] Yu H., Pardoll D., Jove R. (2009). STATs in cancer inflammation and immunity: a leading role for STAT3. *Nature Reviews. Cancer*.

[58] Turkson J. (2004). STAT proteins as novel targets for cancer drug discovery. *Expert Opinion on Therapeutic Targets*.

[59] Kim H. S., Lee M.-S. (2007). STAT1 as a key modulator of cell death. *Cellular Signalling*.

[60] Klover P. J., Muller W. J., Robinson G. W., Pfeiffer R. M., Yamaji D., Hennighausen L. (2010). Loss of STAT1 from mouse mammary epithelium results in an increased Neu-induced tumor burden. *Neoplasia*.

[61] Bowman T., Garcia R., Turkson J., Jove R. (2000). STATs in oncogenesis. *Oncogene*.

[62] Fryknäs M., Dhar S., Öberg F. (2007). STAT1 signaling is associated with acquired crossresistance to doxorubicin and radiation in myeloma cell lines. *International Journal of Cancer*.

[63] Roberts D., Schick J., Conway S. (2005). Identification of genes associated with platinum drug sensitivity and resistance in human ovarian cancer cells. *British Journal of Cancer*.

[64] Weichselbaum R. R., Ishwaran H., Yoon T. (2008). An interferon-related gene signature for DNA damage resistance is a predictive marker for chemotherapy and radiation for breast cancer. *Proceedings of the National Academy of Sciences*.

[65] Wu C., Chen X., Shu J., Lee C.-T. (2017). Whole-genome expression analyses of type 2 diabetes in human skin reveal altered immune function and burden of infection. *Oncotarget*.

[66] Shapiro B., Tocci P., Haase G., Gavert N., Ben-Ze'ev A. (2015). Clusterin, a gene enriched in intestinal stem cells, is required for L1-mediated colon cancer metastasis. *Oncotarget*.

[67] Magrini E., Cavallaro U., Bianchi F. (2015). Microarray profiling of L1-overexpressing endothelial cells reveals STAT3 activation via IL-6/IL-6R*α* axis. *Genomics data*.

[68] Yu H., Zhou P., Li D., Li W. (2019). L1CAM-positive expression is associated with poorer survival outcomes in resected non-small cell lung cancer patients. *International Journal of Clinical and Experimental Pathology*.

